# Editorial: Advances in adolescent vaccination programs in Low- and Middle-Income Countries (LMICs): A focus on HPV vaccination capacity strengthening

**DOI:** 10.3389/fpubh.2022.971578

**Published:** 2022-07-20

**Authors:** Carine Dochez, Benjamin M. Kagina

**Affiliations:** ^1^Network for Education and Support in Immunisation (NESI), Department of Family Medicine and Population Health, Faculty of Medicine and Health Sciences, University of Antwerp, Antwerp, Belgium; ^2^Vaccines for Africa Initiative (VACFA), Faculty of Health Sciences, School of Public Health & Family Medicine, Institute of Infectious Disease and Molecular Medicine, University of Cape Town, Cape Town, South Africa

**Keywords:** adolescents, vaccination, HPV vaccine, Low- and Middle-Income Countries, capacity strengthening

## Introduction

The licensure of the first HPV vaccine was in 2006 and it took nearly a decade for the vaccine to be introduced in LIMCs. At the end of 2021, only 19 African countries had introduced the HPV vaccine in their national immunization programme (1). Globally, impetus to HPV vaccination can be traced to the decade of vaccines (2011–2020) framework that advocated for many issues, among which was adolescent vaccination (2). The momentum to advance HPV vaccination got a boost through the launch of a global strategy to eliminate cervical cancer, outlining several targets that all countries need to meet by 2030 (3). Furthermore, Immunization Agenda (IA) 2030 strategic priority 4 focuses on life course vaccination and integration of other essential health services (4). Adolescent HPV vaccination has the potential to create opportunities to integrate other health-related services such as deworming and vitamin supplementation, more so in LMICs.

In this editorial, we reflect on the key findings from four articles we guest edited for publication in the Research Topic “Advances in adolescent vaccination programs in Low- and Middle-Income Countries (LMICs): A focus on HPV vaccination capacity strengthening”. Our reflections are on three core and related areas all covered in the published articles: vaccine hesitancy, vaccination coverage, and operational challenges related to the delivery of HPV vaccines in LMICs.

## Vaccine hesitancy

In Cameroon, an article by Haddison et al. reported nearly half (50%) of vaccinators being skeptical about HPV vaccination. Although the article reported findings from a small sample size (*n* = 24) of vaccinators in one of the country's districts, negative perceptions of HPV vaccination among the healthcare workers have been reported in many other settings. In many LMICs, healthcare workers are the trusted sources of information related to vaccines and immunization by the caregivers and the public. Therefore, negative perceptions of HPV vaccination by healthcare workers can result to low confidence toward the vaccine by the adolescents which in turn slows the momentum of HPV vaccination programmes. In South Africa, Khosa et al. administered a questionnaire that captured the 5C items to assess HPV vaccine hesitancy among caregivers with girls eligible for vaccination in one of the country's districts with <70% HPV vaccination coverage. The HPV vaccination programme in South Africa was initiated in 2014 at a national level through school-based delivery strategy. The Khosa et al. study reported that nearly 50% of caregivers pointed to factors related to vaccine hesitancy as the barriers to vaccinating their eligible girls. The factors in the 5C model that were found prevalent among the caregivers were: confidence, complacency, constraints, and collective responsibility. Another study in South Africa also pointed out that HPV vaccine hesitancy was observed in caregivers of girls attending private schools (5).

## Vaccination coverage

Low confidence and skepticism toward HPV vaccination by healthcare workers, caregivers and adolescents will lead to suboptimal vaccination coverage. Amponsah-Dacosta et al. reviewed the challenges and opportunities for the HPV vaccination programme in South Africa. The review reports a contrast of >90% coverage observed during the piloting of the programme to a trend of declining HPV vaccination coverage of around 80% in the first year of national rollout and then, as low as around 40% in some districts as the programme matures. The review points to several reasons as potential contributors to the trend in declining HPV vaccination coverage in South Africa: inadequate reminder strategies, poor tracking and targeted follow up for those missing the second dose, weakening social mobilization efforts over time, and inadequate refresher training for vaccinators prior to rolling out the second round of vaccinations. Locally relevant advocacy and communication strategies are needed to address these barriers.

## Operational challenges

For many LMICs, adolescent vaccination was non-existent before HPV vaccine introduction. Operational challenges to HPV vaccine introduction in Kenya were reported in the review published by Karanja-Chege. Among the challenges reported in the review was lack of a reliable estimate for the target number of girls eligible for vaccination, negative sentiments about the HPV vaccines by influential sectors, as well as suboptimal knowledge on the benefits of the vaccination programme by key stakeholders. In South Africa, Amponsah-Dacosta et al. reported challenges related to the supply- and demand-side of the HPV vaccination programme. Supply side challenges include stock availability, costs as well as resources required for delivery of the vaccines. The demand side encompass issues related to vaccine confidence as well as convenient access to the vaccines. Addressing these challenges and taking the lessons learnt forward will be critical in laying a strong foundation for incorporating future adolescent vaccination programmes in LMICs.

## Conclusions

Adolescents, caregivers, healthcare workers, teachers, and the governments are all key stakeholders to advancing the HPV vaccination programmes in LMICs. Therefore, in settings where there is negative perceptions and suboptimal confidence among some of these key stakeholders, public health authorities should promptly act using effective evidence-based interventions to address the concerns of the caregivers and healthcare workers. A possible intervention is to conduct activities, including advocacy and communication strategies, aimed at improving knowledge and addressing low confidence and skepticism toward HPV vaccines. Such activities should be tailor made to the different target groups. For example, HPV vaccination workshops organized in Eastern and Southern Africa contributed to improved knowledge and sharing experiences on HPV vaccination programmes in the region (6). The workshops established an African-based network to advocate for incorporating the HPV vaccine into national immunization programmes, and created a platform for experience exchange that would then foster development of novel ideas of school-based health programmes as delivery platform of adolescent immunization services, as well as identifying ways of reaching out-of-school girls through facility and community based programmes.

Emerging evidence points to a possibility of using a single rather than two dose HPV vaccination schedule (7). Additionally, vaccinology training opportunities aimed at equipping vaccinators and policy makers with improved knowledge to address vaccine hesitancy, low vaccination coverage and operational challenges are increasing. Taken together, these developments will contribute to strengthening of HPV vaccination programmes in LMICs.

## Author contributions

All authors listed have made a substantial, direct, and intellectual contribution to the work and approved it for publication.

## Conflict of interest

The authors declare that the research was conducted in the absence of any commercial or financial relationships that could be construed as a potential conflict of interest.

## Publisher's note

All claims expressed in this article are solely those of the authors and do not necessarily represent those of their affiliated organizations, or those of the publisher, the editors and the reviewers. Any product that may be evaluated in this article, or claim that may be made by its manufacturer, is not guaranteed or endorsed by the publisher.
